# *OsIAA19*, an *Aux*/*IAA* Family Gene, Involved in the Regulation of Seed-Specific Traits in Rice

**DOI:** 10.3390/plants13243538

**Published:** 2024-12-18

**Authors:** Sha-Sha Jia, Xin-Yu Ren, Man-Ni Tong, Si-Yao Jiang, Chang-Quan Zhang, Qiao-Quan Liu, Qian-Feng Li

**Affiliations:** 1 Jiangsu Key Laboratory of Crop Genomics and Molecular Breeding/Zhongshan Biological Breeding Laboratory/Key Laboratory of Plant Functional Genomics of the Ministry of Education, Agricultural College of Yangzhou University, Yangzhou 225009, China; jiashasha0217@126.com (S.-S.J.); renxinyucherry@126.com (X.-Y.R.); tongmanni0814@163.com (M.-N.T.); jiangsiyao202411@163.com (S.-Y.J.); cqzhang@yzu.edu.cn (C.-Q.Z.); qqliu@yzu.edu.cn (Q.-Q.L.); 2 Co-Innovation Center for Modern Production Technology of Grain Crops of Jiangsu Province/Jiangsu Key Laboratory of Crop Genetics and Physiology, Yangzhou University, Yangzhou 225009, China

**Keywords:** *Aux/IAA* gene, *OsIAA19*, auxin, grain shape, chalkiness, seed germination, rice

## Abstract

The Aux/IAA family proteins, key components of the auxin signaling pathway, are plant-specific transcription factors with important roles in regulating a wide range of plant growth and developmental events. The *Aux/IAA* family genes have been extensively studied in Arabidopsis. However, most of the *Aux/IAA* family genes in rice have not been functionally studied. Only two *IAA* genes have been reported to be involved in the regulation of rice grain size. Grain size is a key factor affecting both rice yield and quality. Therefore, we selected an unreported *IAA* member, *OsIAA19*, based on bioinformatics analysis to investigate its potential role in grain size control. Our study showed that *OsIAA19* was constitutively expressed in all tissues tested and that the encoding protein was nuclear localized. The *osiaa19* mutants were then generated using CRISPR/Cas9 gene editing. Agronomic trait analyses showed that the *OsIAA19* mutation significantly increased rice grain length and weight, but had no significant effect on plant height, number of tillers, flag leaf length and width. In addition, the chalkiness of the *osiaa19* mutant seeds also increased, but their eating and cooking quality (ECQ) was not altered. Finally, seed germination analysis showed that knocking out *OsIAA19* slightly suppressed rice seed germination. These results suggest that *OsIAA19* may specifically regulate rice seed-related traits, such as grain shape, rice chalkiness and seed germination. This study not only enriched the functional study of the *Aux/IAA* genes and the auxin signaling pathway in rice, but also provided valuable genetic resources for breeding elite rice varieties.

## 1. Introduction

Phytohormones, trace amounts of organic substances produced by plants, play a key regulatory role in plant growth and development events as well as in adaptation to environmental changes [1]. The phytohormone family mainly includes auxin, gibberellin (GA), cytokinin, ethylene, abscisic acid (ABA), brassinosteroid (BR), jasmonic acid (JA), strigolactone and salicylic acid [2]. Auxin, as the first identified plant hormone, is required for normal plant growth and development processes, such as vascular tissue formation, apical dominance, root formation, tropism, cell division, differentiation and flower and fruit development [3,4,5]. Therefore, auxin is quite important and deserves a lot of attention from scientists.

The elucidation of the biosynthesis and signal transduction mechanism of auxin is of great importance for a better understanding of plant growth and development as well as their response to environmental changes. In general, the synthesis of auxin involves two main pathways, namely the tryptophan-dependent and the tryptophan-independent pathways [6]. Compared to the complex auxin biosynthetic pathways, great progress has been made in elucidating the mechanisms of auxin signaling and transport [2,7]. *TIR1/AFB*-mediated auxin transcriptional regulation, also known as classical auxin signaling, is the best-studied and characterized auxin signaling pathway [8]. This pathway comprises three core transduction components: the auxin receptors, TIR1/AFBs; the transcriptional repressors, Aux/IAA; and the transcription factors, ARFs [9,10,11]. Briefly, in the presence of low levels of auxin, the repressor protein Aux/IAA binds to the active transcription factor ARF to form a heterodimer, which in turn inhibits the transcriptional activating activity of ARF [12]. In contrast, in a high auxin environment, auxin binds to the TIR1/AFB receptor and triggers the degradation of the Aux/IAA proteins via the ubiquitin–proteasome pathway, thereby releasing the ARF transcription factors, which can activate the transcription of auxin-responsive genes [13].

The Aux/IAA protein family is a group of plant-specific transcriptional repressors that are mainly involved in the regulation of root and leaf formation, phototropism, gravitropism, and apical dominance of buds [14]. There are a total of 29 members of the *Aux/IAA* gene family in Arabidopsis thaliana, distributed on five chromosomes [15]. Due to the similarity of IAA proteins, the knock-out of some genes in the *Aux/IAA* family has little effect on the plant phenotype, indicating the existence of functional redundancy among some *Aux/IAA* members [16]. Therefore, the functions of many *Aux/IAA* genes in Arabidopsis have been characterized using the gain-of-function mutation strategy [17,18,19,20,21,22].

Rice is not only a model monocotyledonous plant, but also one of the most important food crops in the world. More than half of the world’s population feeds on rice. The production of elite rice varieties with superior quality and high yield is the main goal of rice breeding. Given the critical role of auxin in plants, extensive functional studies of rice *Aux/IAA* genes are essential. According to phylogenetic sequence analysis, there are 31 members of the *Aux/IAA* gene family in rice [23]. However, most of the *Aux/IAA* genes are uncharacterized and their biological functions are still unclear.

To date, the *Aux/IAA* genes reported in rice include *OsIAA1*, *OsIAA3*, *OsIAA4*, *OsIAA6*, *OsIAA10*, *OsIAA11*, *OsIAA12*, *OsIAA13*, *OsIAA18*, *OsIAA20*, *OsIAA23* and *OsIAA31.* Of these identified *Aux/IAA* genes, almost half are involved in the control of rice root formation. In particular, overexpression of *OsIAA1* resulted in reduced inhibition of root elongation in response to auxin treatment [24]. Similarly, overexpression of *OsIAA31* resulted in an auxin-insensitive phenotype, including a reduced number of coronal roots, abnormal gravity of roots and aboveground parts [25]. Regarding the regulation of lateral root development, different *Aux/IAA* genes have different or even opposite effects. For example, a gain-of-function mutation in *OsIAA11* inhibits lateral root initiation in rice [26]. On the other hand, the mutation of either *OsIAA13* or *OsIAA23* leads to a reduction in the number of lateral roots [27,28]. In addition, some *Aux/IAA* genes are also involved in the regulation of plant architecture [29]. For example, overexpression of *OsIAA1* reduced plant height and loosened plant architecture [24]. Similarly, overexpression of *OsIAA4* resulted in reduced plant height, increased tiller and leaf angles, and reduced gravity response, although expression level is low throughout the life cycle of rice [30]. *OsIAA6* controls the number of tillers by inhibiting the growth of tiller buds, while *OsIAA12* plays a positive role in the control of leaf angle in rice [31,32]. In addition, *Aux/IAA* genes can also mediate rice responses to abiotic stresses [33]. *OsIAA6* can induce an auxin-mediated drought response by regulating the expression of auxin synthesis genes, and its overexpression enhances drought resistance [31]. *OsIAA18* positively regulates drought and salt stress responses in plants by modulating stress-induced ABA signaling [34]. *OsIAA20* is also a positive regulator of drought tolerance and salt tolerance in rice [35]. Finally, only two *Aux/IAA* genes have been reported to function in the regulation of rice grain size. In more detail, the knock-down of *OsIAA3* resulted in longer rice grains [36]; conversely, overexpression of *OsIAA10* increased grain length and grain weight [37], suggesting that *OsIAA3* is a negative regulator of rice grain length, whereas *OsIAA10* is a positive regulator of rice grain length.

Grain size, a key agronomic trait closely related to both rice yield and quality, has attracted considerable attention from rice geneticists and breeders. To date, more than 80 grain size-related genes have been cloned in rice, and a number of regulatory mechanisms have been identified, including the G-protein pathways, the ubiquitin–proteasome pathway, the mitogen-activated protein kinase (MAPK) signaling pathway, and plant hormones [38]. Within the plant hormone family, BR is known to be an important positive regulator of grain size in rice, and a number of BR-biosynthetic or signaling components are involved in controlling grain size [39]. With respect to auxin, the research progress on rice grain size regulation is still lagging behind. For example, although the number of functionally dissected *Aux/IAA* genes in rice is increasing, the functions of most members have not yet been identified so far. Furthermore, as mentioned above, only *OsIAA3* and *OsIAA10* are involved in grain size control [36,37].

To further identify novel grain size regulators in the *Aux/IAA* gene family, we first performed a phylogenetic analysis of the *Aux/IAA* genes. Then, using expression pattern assay, we successfully identified a potential target, *OsIAA19*, which is an unreported gene with relatively high expression in rice panicles and seeds. We speculated that *OsIAA19* may play an important role in controlling grain size and other seed-related traits. Therefore, a series of analyses were then conducted in this study to investigate the expression and biological functions of *OsIAA19*, including bioinformatics analysis, expression pattern assay and examination of rice yield or grain quality related key agronomic traits of *osiaa19* mutants. Our study not only revealed the biological function of *OsIAA19* and improved the auxin signaling pathway in rice, but also provided a valuable gene resource for rice grain size improvement.

## 2. Results

### 2.1. Bioinformatic Analysis of the Aux/IAA Gene Family in Rice

**Figure 1:**
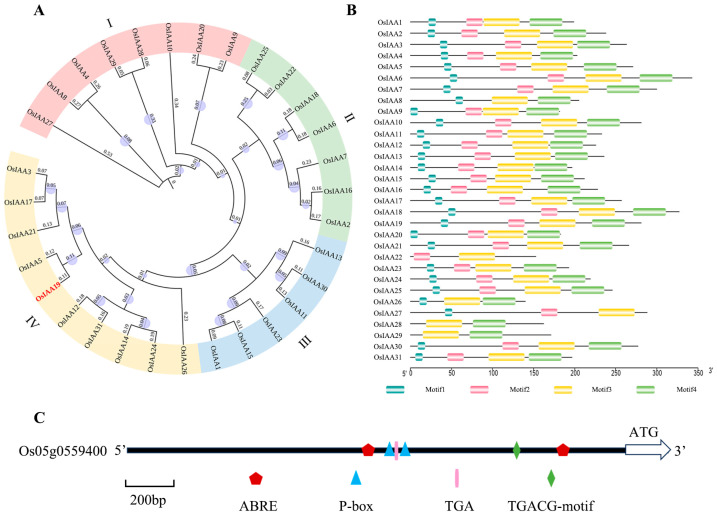
Bioinformatic analysis of the Aux/IAA protein family and the *OsIAA19* promoter in rice. (**A**) Phylogenetic tree of the Aux/IAA protein family in rice. The tree was constructed using the adjacency method in the MEGA7 software. The bootstrap is set to 1000. (**B**) Conserved motif analysis of the OsIAA protein in rice. (**C**) Analysis of hormone and stress response elements in the 2 kb upstream region of the *OsIAA19* transcription start site (ATG). P-box is a gibberellin response element. ABRE is an ABA response element. TGACG is related to MeJA and TGA is an auxin response element.

First, we constructed the phylogenetic tree of the *OsIAA* gene family in rice using MEGA7 software [40], which divided the whole family into four subfamilies according to the degree of evolution (Figure 1A). Subfamilies I to IV contain eight, seven, six and ten genes, respectively. The conserved motifs of the Aux/IAA family were analyzed using the MEME website [41]. The result showed that 25 out of 31 members shared four conserved motifs (Figure 1B). Subfamily IV had the largest number of members, but only three of the 10 members have been reported so far. Interestingly, the subfamily includes *OsIAA3*, the negative regulator of rice grain. In order to isolate target genes with a potential role in grain size regulation, the ePlants Rice website [42] was used to predict the expression patterns of the uncharacterized genes in this branch. The result showed that the expression level of *OsIAA19* in panicle and seed was significantly higher than that of the other genes (Supplementary Figure S1A). To further confirm their expression patterns, RT-qPCR analysis was performed. The data showed that *OsIAA17* had the highest expression in young rice panicles, while the expression of *OsIAA19* was close to that of *OsIAA17*. The expression of the other five genes was quite low (Supplementary Figure S1B). A similar result was obtained in the expression analysis of developing seeds. A major difference is that *OsIAA19* had the highest expression, which was about two times higher than that of *OsIAA17*, which had the second highest expression level (Supplementary Figure S1C), suggesting that *OsIAA19* may be involved not only in the regulation of rice grain size, but also in the control of grain filling or other seed-related traits. Therefore, *OsIAA19* was selected for the following expression and functional analysis.

The full-length cDNA of the *OsIAA19* gene is 1261 bp, contains 5 exons and encodes a 281-amino-acid Aux/IAA protein. We then analyzed the promoter of *OsIAA19* by using PlantCARE [43]. The result showed that the *OsIAA19* promoter contained a number of motifs related to plant hormones, including two P-box elements, two ABRE motifs, a TGACG motif and a TGA motif (Figure 1C). In particular, the P-box element is involved in the GA response and the ABRE element is involved in the ABA response. The TGACG motif and the TGA element are involved in the ME-JA response and the auxin response, respectively. The information suggested that the *OsIAA19* gene may be involved in several plant hormone pathways. At present, studies have confirmed that genes related to GA and auxin pathways, such as *GW6* and *OsARF6*, are involved in the regulation of rice grain size [38]. However, no such genes have been reported in the ABA and MEJA pathways.

### 2.2. The OsIAA19 Gene Has a Constitutive Expression Pattern and Its Protein Is Localized in the Nucleus

**Figure 2:**
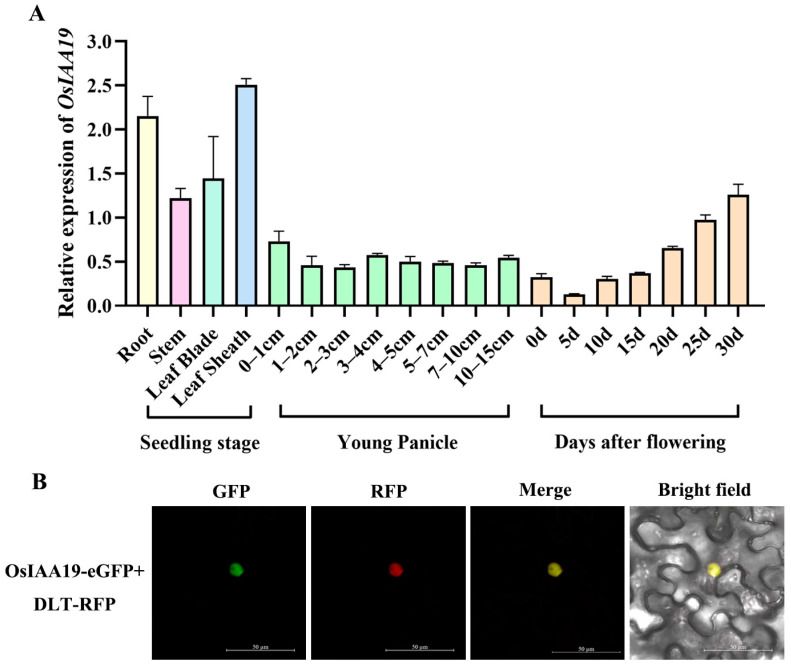
Expression analysis of *OsIAA19*. (**A**) The expression of *OsIAA19* was analyzed in different tissues, including developing spikelets and seeds. *Actin01* was used as an internal control for normalization. Data are means ± SD (n = 3 biological replicates). (**B**) Co-localization analysis of OsIAA19-eGFP protein and DLT-RFP protein in tobacco epidermal cells. The scale bar is 50 μm.

To clarify the actual spatial and temporal expression pattern of the *OsIAA19* gene, total RNA was extracted from various tissues as well as developing panicles and seeds at different stages of wild-type rice ZH11. After the corresponding cDNA was generated by reverse transcription, the expression pattern of *OsIAA19* was analyzed by RT-qPCR. The results showed that *OsIAA19* was expressed in all rice samples tested, including roots, stems, leaves and leaf sheaths at the heading stage, as well as developing spikelets and seeds, indicating that the *OsIAA19* gene has a constitutive expression pattern (Figure 2A). More specifically, the expression abundance of *OsIAA19* was maintained throughout the developmental stages of spikelet tissues. However, the expression of the *OsIAA19* gene gradually increased with the development of rice seeds (Figure 2A), suggesting that OsIAA19 plays multiple roles in the development of rice spikelets and seeds.

To further investigate the subcellular localization of the OsIAA19 protein, the *OsIAA19* gene sequence was fused with the *eGFP* coding gene and transformed into the tobacco epidermal cells. The GFP fluorescence signal was then observed using laser confocal microscopy. The result showed that the OsIAA19 protein was only localized in the nucleus, whereas the eGFP protein alone was present in both the cytoplasm and the nucleus (Supplementary Figure S2). To further confirm the nuclear localization of OsIAA19, a nuclear-specific marker DLT protein was selected for co-localization assay [44]. DLT was fused to the RFP protein and co-expressed with the OsIAA19-eGFP protein. The results showed that OsIAA19-eGFP and DLT-RFP co-localized well in the nucleus (Figure 2B), demonstrating that OsIAA19 is a nuclear-localized protein.

### 2.3. Knockout of OsIAA19 Had Little Effect on Rice Morphology

**Figure 3:**
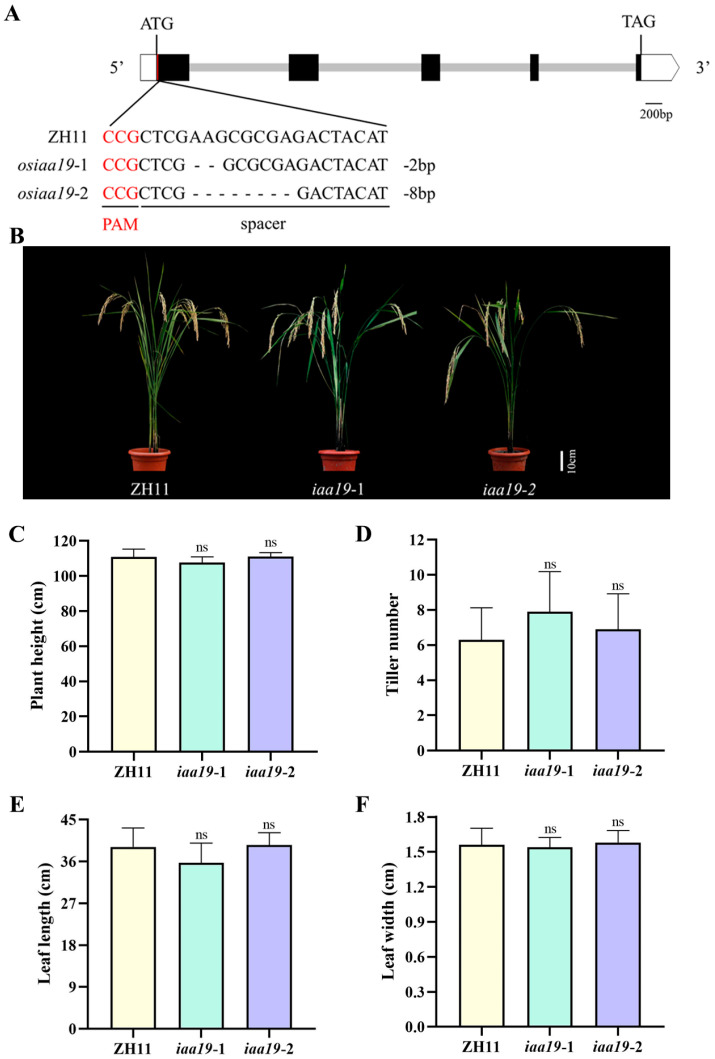
Analysis of key agronomic traits of *osiaa19* mutants and the wild-type control. (**A**) Schematic diagram of the vector used for CRISPR/Cas9-mediated gene editing of *OsIAA19*. The target site is underlined and the protospacer adjacent motif (PAM) is highlighted in red. Deletions are indicated by hyphens. (**B**) Morphology of *osiaa19* mutants and wild type ZH11. The scale bar is 10 cm. Quantitative data of plant height (**C**), number of tillers (**D**), leaf length (**E**) and leaf width (**F**) of the *osiaa19* mutants and wild-type control. Data are means ± SD (n = 5 rice plants). ns, not significant (Student’s *t*-test).

To further investigate the biological function of *OsIAA19*, CRISPR/Cas9 gene editing technology was used to generate *osiaa19* rice mutants. The editing target site of the *OsIAA19* gene in the generated transgenic rice was then confirmed by sequencing and finally, two homozygous *osiaa19* mutants were obtained, named *osiaa19-1* and *osiaa19-2*, respectively (Supplementary Figure S3). Specifically, two bases were deleted in the target region of *OsIAA19* in mutant *osiaa19-1*, and eight bases were deleted in mutant *osiaa19-2*, both resulting in frameshift mutations (Figure 3A). We then examined the main agronomic traits of the *osiaa19* mutants, including plant height, number of tillers, leaf length and width. The analysis showed that no significant difference was observed between the *osiaa19* mutants and the wild-type control ZH11 in these traits (Figure 3B–F), indicating that the *OsIAA19* mutation had no effect on normal plant growth.

### 2.4. OsIAA19 Mutation Increased Rice Grain Length and Weight

**Figure 4:**
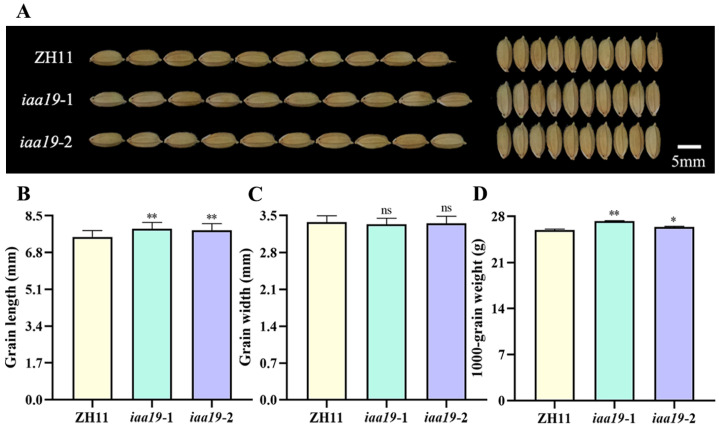
Grain size analysis of *osiaa19* mutants and ZH11 control. Grain morphology (**A**), grain length (**B**), grain width (**C**) and 1000-grain weight (**D**) of *osiaa19* mutants and ZH11 control were analyzed. Data are means ± SD (n = 100 seeds). * *p* < 0.05; ** *p* < 0.01; ns, not significant (Student’s *t*-test).

Since the regulation of grain size is important for both rice yield and grain quality, we then analyzed the grain shape of both *osiaa19* mutants and wild type (Figure 4A). The results showed that the grain length of the *osiaa19* mutants was significantly longer than that of the ZH11 control (Figure 4A,B). There was no difference in grain width between *osiaa19* mutants and wild type (Figure 4C). As a consequence, the 1000-grain weight of the *osiaa19* mutants was significantly increased (Figure 4D). Similarly, the glume length of the *osiaa19* mutants was also increased at the heading stage, while the width remained unchanged (Supplementary Figure S4).

**Figure 5:**
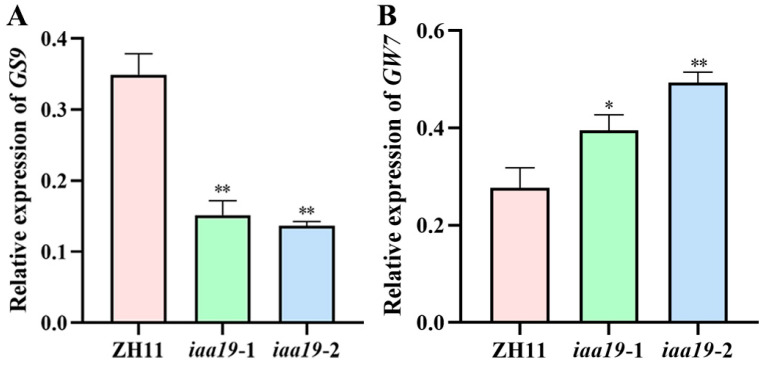
Expression analysis of the grain size genes *GS9* (**A**) and *GW7* (**B**) in rice spikelets of *osiaa19* mutants and ZH11 control. *Actin01* was used as an internal control for gene expression. Data are means ± SD (n = 3 biological replicates). * *p* < 0.05; ** *p* < 0.01 (Student’s *t*-test).

To preliminarily explore the potential mechanism of *OsIAA19* in regulating rice grain length, we examined the expression of two representative grain size genes, *GS9* [45] and *GW7* [46]. The results showed that the expression of *GS9*, a negative regulator of grain length, was significantly reduced in *iaa19* mutants (Figure 5A). In contrast, the expression of *GW7*, a positive regulator of grain length, was significantly increased in *iaa19* mutants (Figure 5B). These data suggest that *OsIAA19* negatively regulates rice grain length, at least in part, by modulating the expression of *GS9* and *GW7*.

### 2.5. OsIAA19 Mutation Increased Rice Chalkiness but Without Significant Effect on Eating and Cooking Quality (ECQ)

**Figure 6:**
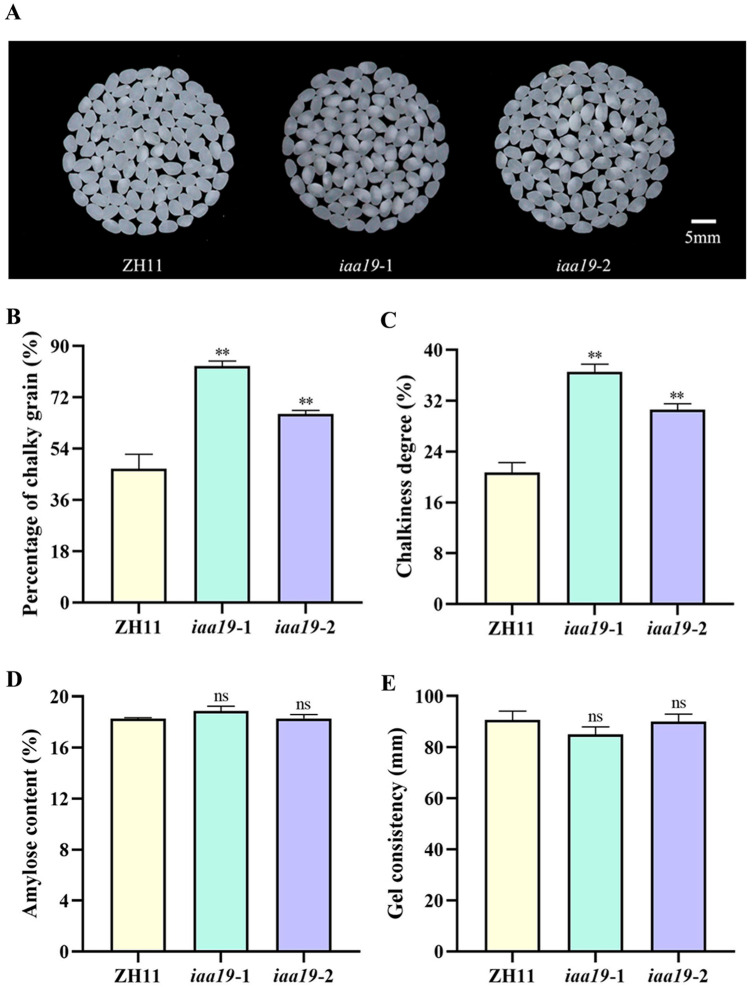
Analysis of the appearance quality and physicochemical properties of the *osiaa19* mutants and the wild type ZH11. (**A**) The appearance of the milled rice from *osiaa19* mutants and ZH11. Chalky grain rate (**B**), chalkiness degree (**C**), AC (**D**), and GC (**E**) of the *osiaa19* mutants and ZH11 control. Data are means ± SD (n = 3 biological replicates). In each biological replicate, 150–200 grains were used for seed chalkiness analysis. ** *p* < 0.01; ns, not significant (Student’s *t*-test).

Chalkiness refers to opaque areas in the endosperm of rice that reduce the processing and appearance quality of rice [47]. The degree of chalkiness can be measured by two indices: chalky grain rate and chalkiness degree [48]. The result showed that both the chalky grain rate and chalkiness degree of the *osiaa19* mutants were significantly increased (Figure 6A–C). In addition to rice appearance, two important physicochemical properties of rice, amylose content (AC) and gel consistency (GC), were also investigated. AC is the most important determinant of rice ECQ, and GC is another important index for evaluating rice ECQ. The data showed that both the AC and GC of *osiaa19* mutants were not significantly changed compared to the control ZH11 (Figure 6D,E).

### 2.6. OsIAA19 Mutation Slightly Suppresses Seed Germination

**Figure 7:**
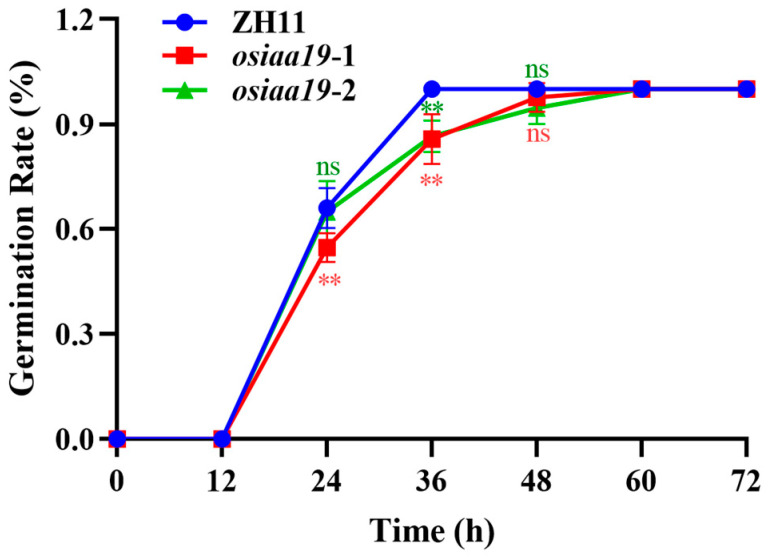
Seed germination analysis of the *osiaa19* mutants and the wild-type control ZH11. Data are means ± SD (n = 3 biological replicates). In each biological replicate, 30 seeds were used for analysis. ** *p* < 0.01; ns, not significant (Student’s *t*-test).

The ability of rice seeds to germinate is critical for direct seeding and ensuring high rice yields. Since OsIAA19 is a component of the auxin signaling pathway and a number of other hormone-related cis-elements exist in its promoter, we further investigated whether the *OsIAA19* mutation would affect the germination property of rice seeds. The results showed that the germination rate of the *osiaa19* mutants was slower than ZH11 at the early stage of germination, but the final germination rate was not affected (Figure 7). In addition, the bud length of the *osiaa19* mutants was shorter (Supplementary Figure S5).

## 3. Discussion

The study of rice grain size is of great importance for the cultivation of rice varieties with high yield and superior quality. Grain size includes the length, width and thickness of grains, as well as the ratio of length to width. Grain size not only affects rice yield but also the appearance quality and processing quality of rice [49,50,51]. More than 80 rice grain shape genes have been cloned, and a number of different regulatory mechanisms have been successfully revealed [52,53]. Among them, plant hormones are important regulators of rice grain size. Recently, a number of publications have reported that the plant hormones BR, auxin, GA and cytokinin play important roles in regulating grain size [38].

Auxin plays an important role in a wide range of growth and developmental events in rice. Several auxin-related genes have been reported to regulate grain size in rice. *BG1*, a primary response gene of auxin, functions as a positive regulator of auxin response and transport and consequently regulates grain size by modulating cell division and elongation [54]. *TGW6* encodes indole-3-acetic acid (IAA)-glucose hydrolase for the production of free IAA. Knocking out *TGW6* increases grain length and weight [55]. Further studies showed that *TGW6* is expressed for only a short time during early inflorescence development, suggesting that *TGW6* may also play an important role in regulating pollen development [56]. *qTGW3* encodes a GSK3/SHAGGY-like kinase OsSK41/OsGSK5 that interacts with and phosphorylates the transcriptional repressor OsARF4 in the auxin pathway. The OsSK41–OsARF4 interaction module may repress rice grain size and weight by negatively regulating auxin signaling [57]. In addition, a recent study revealed a novel mechanism of grain size regulation centered on the OsTIR1–OsIAA10–OsARF4 module [37]. Finally, as a transcription factor, OsARF6 binds directly to the promoter of the auxin influx transporter gene *OsAUX3* to negatively regulate rice grain length and weight. Mechanistically, the OsARF6–OsAUX3 module affects the longitudinal elongation of glume cells by altering both the level and distribution of auxin in glume cells. In addition, miR167a can directly direct OsARF6 mRNA silencing to increase rice grain length and weight [58].

There are a total of 31 *Aux/IAA* gene members in rice. However, only two of them, *OsIAA3* and *OsIAA10*, have been reported to be involved in grain size regulation. Interestingly, the two genes play opposite roles in controlling grain size. Specifically, *OsIAA3* is a negative regulator, whereas *OsIAA10* is a positive regulator of grain size [36,37]. In this study, we demonstrated that, like *OsIAA3*, *OsIAA19* also functions as a negative regulator of rice grain size because the knock-out of *OsIAA19* promoted rice grain length and weight (Figure 4). In addition, our data showed that *OsIAA19* mutation had no significant effect on rice plant height, tiller, leaf length and width (Figure 3). Therefore, *OsIAA19* is considered to be a novel gene with a specific regulatory role in seed-related traits, including grain size. However, the underlying molecular mechanism still requires further investigation.

In addition to grain size, some other seed-related traits of the *osiaa19* mutants were also investigated, including rice quality and germination characteristics. In general, rice quality includes processing quality, appearance quality, eating and cooking quality, and nutritional quality. Appearance quality is mainly influenced by grain shape, chalkiness and transparency [38]. In addition, grain shape is closely related to chalkiness. In general, the slender grain, i.e., with longer grain length and narrower grain width, usually has a better appearance quality with less chalkiness. For example, *GW2* encodes an E3 ubiquitin ligase that negatively affects grain width and grain weight in rice [59]. NIL-*gw2.1*, the near-isogenic line of *GW2*, not only increased grain length and width, but also reduced chalkiness [60]. OsSPL16/GW8, a transcription factor containing the SBP domain, can bind directly to the promoter of *GW7* and inhibit its expression, thereby regulating rice grain width [61,62]. In addition, phytohormones are also involved in the regulation of chalkiness. *DG1* is mainly responsible for the long-distance transport of ABA from rice leaves to grains, and its mutation resulted in abnormal grain filling and subsequent silty endosperm [63]. OsNF-YB1, an endosperm-specific transcription factor, binds to the promoter of *OsYUC11* and positively regulates auxin synthesis. Therefore, IAA biosynthesis was reduced in *osyuc11* and *osnf-yb1* mutants, corresponding to smaller seeds and increased chalkiness [64]. Surprisingly, although grain length increased in *osiaa19*, grain chalkiness also increased (Figure 6). However, it is still unclear whether the increased chalkiness in the *osiaa19* mutants is caused by a change in grain size or a grain filling problem, b66ecause *OsIAA19* was also highly expressed in rice source tissues such as rice leaf. In fact, GWD1, an enzyme involved in controlling transient starch degradation in source tissues, also plays an important regulatory role in seed traits such as grain size, rice chalkiness and seed germination [65], suggesting the importance of source–sink interactions in determining important seed traits.

## 4. Materials and Methods

### 4.1. Bioinformatic Analysis

According to the gene IDs of 31 members of the *Aux/IAA* family in rice (Supplementary Table S1), their corresponding protein sequences were used to construct the phylogenetic tree by using the MEGA7 software with the neighbor-joining method [66], and the bootstrap was set to 1000. The constructed phylogenetic tree was then further refined by ITOL (https://itol.embl.de/, accessed on 30 October 2024). The conserved motifs of the Aux/IAA family were analyzed using the MEME websites (https://meme-suite.org/meme/, accessed on 30 October 2024).

### 4.2. Plant Materials and Growth Conditions

The rice materials used in this study were *osiaa19* mutants and their corresponding wild-type control japonica rice variety Zhonghua 11 (ZH11). All rice materials were grown in the field of the Agricultural College of Yangzhou University. During the planting process, each experimental material was planted in two rows according to the standard wide and narrow rows, with ten seedlings in each row, and the conventional crop management methods were followed for uniform management.

### 4.3. Vector Construction

We designed specific targets for gene editing in the first exon of the *OsIAA19* gene. The target DNA sequence was then cloned into the sgRNA-Cas9 expression vector. After confirmation by sequencing, the correct target plasmid was transformed into rice callus by the Agrobacterium-mediated transformation method. The CRISPR/Cas9-related vector system and the detailed procedure can be found in a previous publication [67]. The primers used for sequencing are shown in Supplementary Table S2.

### 4.4. Agronomic Trait Analysis

The agronomic traits of the *osiaa19* mutants and the wild-type control ZH11 were investigated in the experimental field at Yangzhou University. The agronomic traits investigated included plant height, number of tillers, flag leaf length and width. As to the seed-related traits, grain length, grain width and 1000-grain weight were measured using the rice appearance quality detector (SC-E, Wanshen, Hangzhou, China).

### 4.5. RT-qPCR Assay

Samples of different rice tissues and developing spikelets or seeds were collected for *OsIAA19* gene expression analysis. Total RNA was carefully extracted from the above samples using an RNA extraction kit (RC401, Vazyme, Nanjing, China). Then, 1 μg of total RNA was used for reverse transcription. Finally, RT-qPCR analysis was performed using the 2 × ChamQ SYBR qPCR Master Mix Kit (Q711, Vazyme, Nanjing, China), and the *Actin01* gene was used as a reference gene for normalization. Three biological replicates were used in each experiment. All primer sequences used are listed in Supplementary Table S2.

### 4.6. Subcellular Localization Analysis

To study the subcellular localization of the OsIAA19 protein, the full-length OsIAA19 CDS sequence was ligated into the p2300-35S-eGFP vector to construct the 35S::OsIAA19-GFP plasmid, then transfected into the Agrobacterium strain GV3101 for transient expression in tobacco leaf epidermal cells. DLT-RFP, as a nuclear localization marker, was co-expressed with OsIAA19-eGFP in tobacco leaf epidermal cells. Finally, the GFP and RFP signals in the transferred tobacco leaves were observed by using the laser confocal microscope (LSM 710, Carl Zeiss AG, Jena, Germany).

### 4.7. Rice Flour Preparation and Rice Appearance Analysis

First, a rice hulling machine (SY88-TH, SsangYong, Hirazawa, Republic of Korea) was used to dehull the ripe seeds to obtain brown rice. Then, the brown seeds were manually selected to remove the moldy and incompletely matured seeds. Next, the brown rice was polished using a grain polisher (Pearlest, KETT, Tokyo, Japan) to obtain polished rice [68]. After removing the broken rice, the whole milled rice was scanned by the rice appearance quality detector (SC-E, Wanshen, Hangzhou, China) to evaluate the chalky rice.

### 4.8. Rice Quality Analysis

The rice flour prepared above was used to analyze the amylose content (AC) and gel consistency (GC) of rice. The detailed procedure could follow the previous publication [69].

### 4.9. Seed Germination Test

The mature rice seeds were manually hulled, sterilized and incubated in the dark in an artificial climate incubator at a temperature of 26 °C for seed germination analysis. A detailed method of seed germination testing can be found in previously published literature [70].

### 4.10. Statistical Analysis

The data were analyzed using GraphPad Prism 8.0.2. Data in this study were presented as mean ± SD. The level of significance was assessed using Student’s *t*-test (* *p* < 0.05, ** *p* < 0.01).

## 5. Conclusions

In this study, we performed a bioinformatic analysis of the Aux/IAA family proteins. Based on the phylogenetic tree data and the expression pattern of *OsIAA* genes, *OsIAA19* was selected as a candidate gene for further analysis of its role in controlling rice grain size. First, expression analysis showed that the *OsIAA19* gene was constitutively expressed in all tissues tested, including the developing spikelets. Next, the subcellular localization assay showed that the OsIAA19 protein was specifically localized in the nucleus. *osiaa19* mutants were then generated using CRISPR/Cas9 gene editing. Agronomic trait analysis showed that, except for increased grain length, the other rice traits tested were unchanged. In addition, grain quality analysis showed that the *OsIAA19* mutation had no effect on rice ECQ, but increased grain chalkiness. Finally, the germination of *osiaa19* mutants was slightly inhibited. In conclusion, this research not only dissected the expression pattern and biological function of a new *Aux/IAA* gene *OsIAA19* in rice, but also provides useful information on the regulatory network of the auxin pathway and is an important gene resource for future rice quality improvement.

## Data Availability

All data supporting the findings of this study are available within the paper and Supplementary Materials.

## Supplementary Materials

The following supporting information can be downloaded at: https://www.mdpi.com/article/10.3390/plants13243538/s1, Supplementary Figure S1: Prediction of the expression pattern of *OsIAA* genes; Supplementary Figure S2: Subcellular localization of OsIAA19 protein in tobacco epidermal cells; Supplementary Figure S3: Sequencing data of *osiaa19* mutants; Supplementary Figure S4: Morphology of spikelet hulls of *osiaa19* mutants and ZH11 control at heading stage; Supplementary Figure S5: Photographs of germinating seeds 96 h after imbibition (HAI); Supplementary Table S1: Gene IDs of *OsIAA* family members in rice; Supplementary Table S2: Sequence of the primers used in the study.
