# Supplementary material for: OsIAA19, an Aux/IAA Family Gene, Involved in the Regulation of Seed-Specific Traits in Rice
Source: Plants (Basel). 2024 Dec 18;13(24):3538. doi: 10.3390/plants13243538 (PMC11678818; doi:10.3390/plants13243538)
Supplement: Supplementary file 1 [file plants-13-03538-s001.zip › plants-3338593-supplementary.pdf]

OsIAA19, an *Aux/IAA* family gene, involved in the regulation of seed-specific traits in rice

Sha-Sha Jia[1,3], Xin-Yu Ren[1,3], Man-Ni Tong[1], Si-Yao Jiang[1], Chang-Quan Zhang[1,2], Qiao-Quan Liu[1,2], Qian-Feng Li[1,2*]

Supplementary Materials

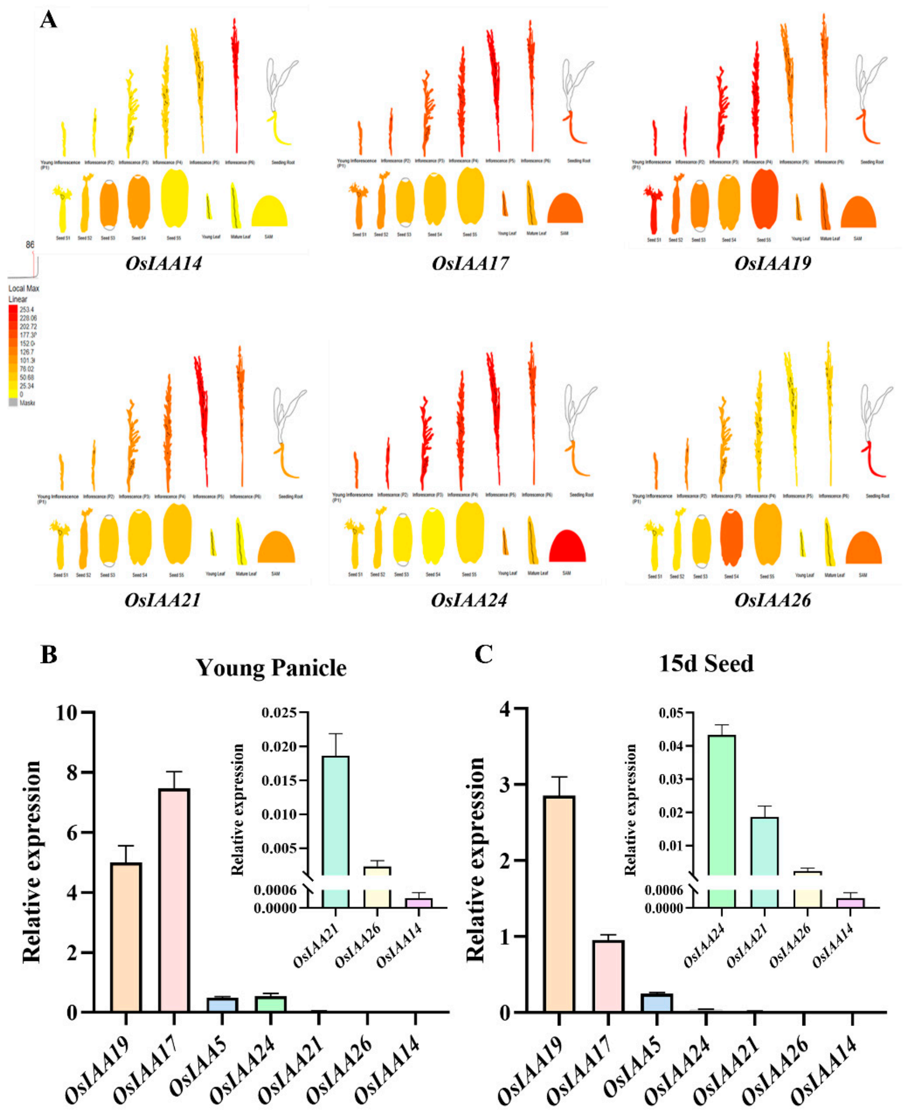

Supplementary Figure S1. Expression analysis of *OsIAA5*, *OsIAA14*, *OsIAA17*, *OsIAA19*, *OsIAA21*, *OsIAA24* and *OsIAA26* in both young panicles (B) and developing seeds 15 d after flowering (C). *Actin01* was used as an internal control for gene expression. Data are means ± SD (n=3 biological replicates).

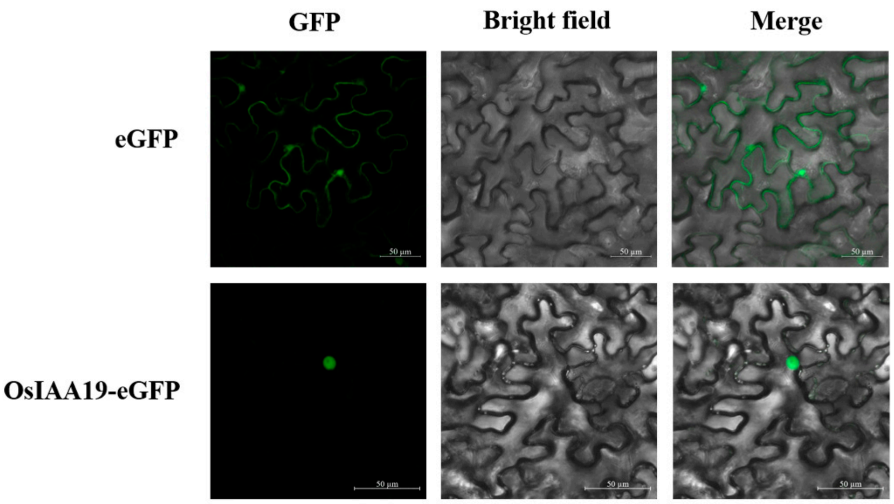

Supplementary Figure S2. Subcellular localization analysis of OsIAA19-eGFP protein in tobacco epidermal cells. eGFP protein alone is also expressed in tobacco epidermal cells as a control. The scale bar is 50 μm.

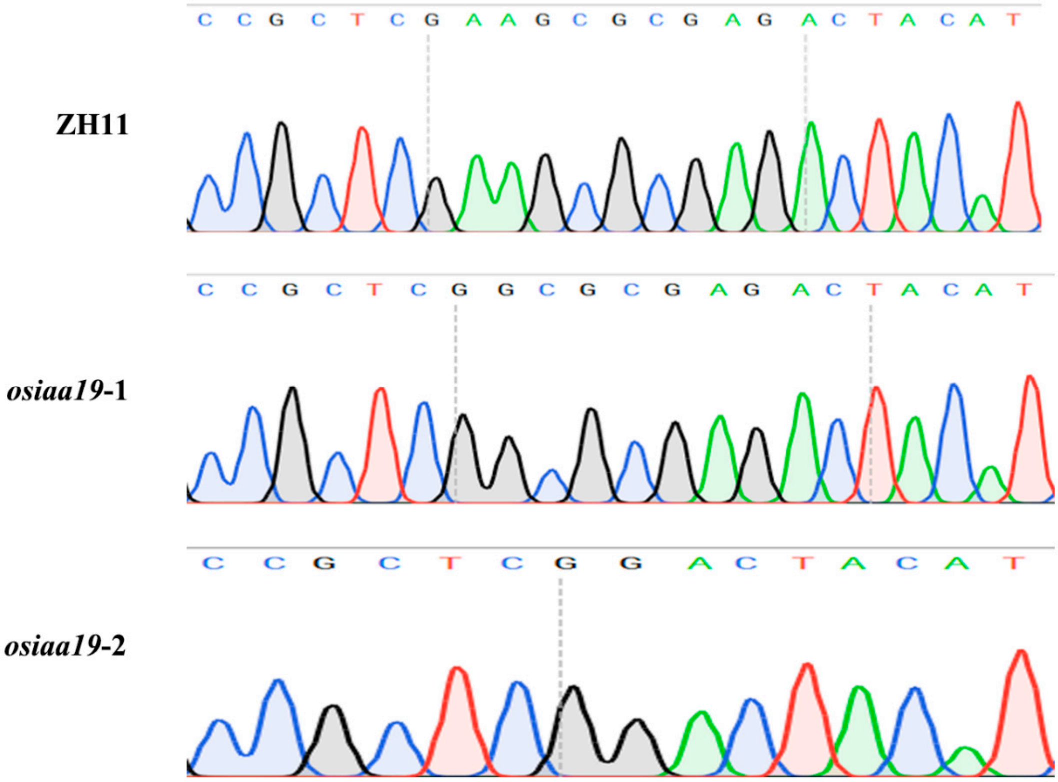

Supplementary Figure S3. Sequencing data of *osiaa19* mutants.

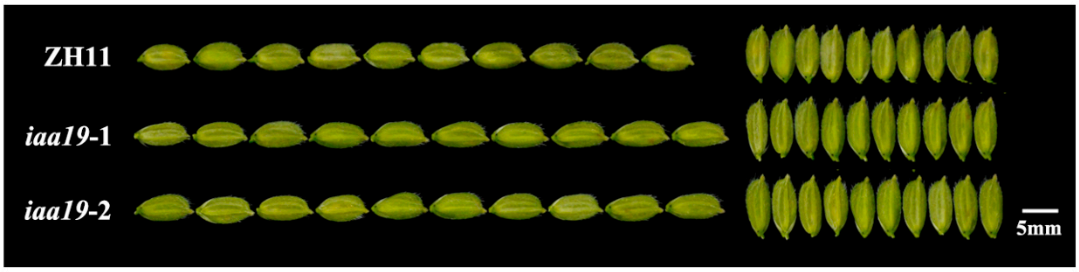

Supplementary Figure S4. Morphology of *osiaa19* mutant and ZH11 control spikelets at heading stage. Scale bar, 5 mm.

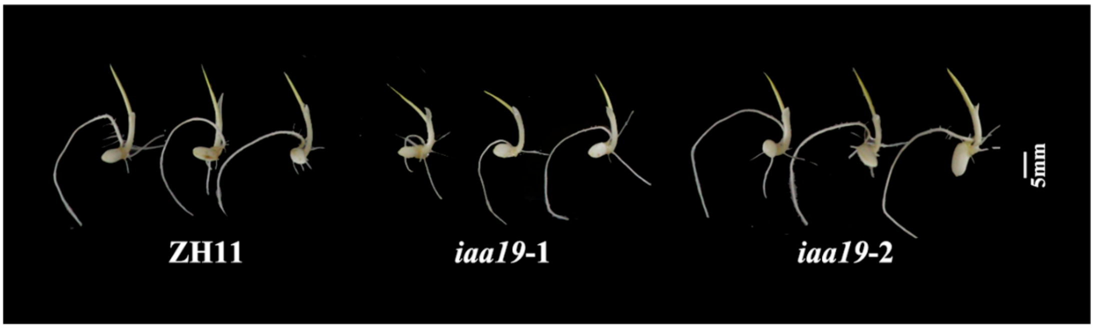

Supplementary Figure S5. Photographs of germinating seeds 96 h after imbibition (HAI).

Supplementary Table S1. Gene IDs of *OsIAA* family members in rice.

Gene ID	Gene name	Gene ID	Gene name	Gene ID	Gene name	Gene ID	Gene name
LOC_Os01g08320	*OsIAA1*	LOC_Os02g56120	*OsIAA9*	LOC_Os05g14180	*OsIAA17*	LOC_Os08g01780	*OsIAA25*
LOC_Os01g09450	*OsIAA2*	LOC_Os02g57250	*OsIAA10*	LOC_Os05g44810	*OsIAA18*	LOC_Os09g35870	*OsIAA26*
LOC_Os01g13030	*OsIAA3*	LOC_Os03g43400	*OsIAA11*	LOC_Os05g48590	*OsIAA19*	LOC_Os11g11410	*OsIAA27*
LOC_Os01g18360	*OsIAA4*	LOC_Os03g43410	*OsIAA12*	LOC_Os06g07040	*OsIAA20*	LOC_Os11g11420	*OsIAA28*
LOC_Os01g48450	*OsIAA5*	LOC_Os03g53150	*OsIAA13*	LOC_Os06g22870	*OsIAA21*	LOC_Os11g11430	*OsIAA29*
LOC_Os01g53880	*OsIAA6*	LOC_Os03g58350	*OsIAA14*	LOC_Os06g24850	*OsIAA22*	LOC_Os12g40890	*OsIAA30*
LOC_Os02g13520	*OsIAA7*	LOC_Os05g08570	*OsIAA15*	LOC_Os06g39590	*OsIAA23*	LOC_Os12g40900	*OsIAA31*
LOC_Os02g49160	*OsIAA8*	LOC_Os05g09480	*OsIAA16*	LOC_Os07g08460	*OsIAA24*		

Supplementary Table S2. Sequence of the primers used in the study

Primer Name	Primer Sequences (5'-3')	Usage
OsActin-qRT-F	CCAAGGCCAATCGTGAGAAGAT	RT-qPCR
OsActin-qRT-R	AATCAGTGAGATCACGCCCCAG	RT-qPCR
OsIAA5-qRT-F	CCTCCAAGACCAAGAGTATGTC	RT-qPCR

OsIAA5-qRT-R	TTCCGGCAGATAGTGGTAAC	RT-qPCR
OsIAA14-qRT-F	GGAGCACGTTCCAGTCTTCGT	RT-qPCR
OsIAA14-qRT-R	GCCGTCCTTGTCCTCATAGGC	RT-qPCR
OsIAA17-qRT-F	GGATGAGGATGCGGAGGA	RT-qPCR
OsIAA17-qRT-R	GTAGCTGCGAATTGGTGGC	RT-qPCR
OsIAA19-qRT-F	AGGTTAGCATGGATGGAGCC	RT-qPCR
OsIAA19-qRT-R	TCCCAAGGAAGATCACCAACA	RT-qPCR
OsIAA21-qRT-F	CCTGCTTTGGCGTATGTATC	RT-qPCR
OsIAA21-qRT-R	ATGGCATGGAGAATTGTCTG	RT-qPCR
OsIAA24-qRT-F	GGCACTGGATCTCCTCTTCA	RT-qPCR
OsIAA24-qRT-R	TCAGACCATCACCTGGCTTC	RT-qPCR
OsIAA26-qRT-F	CTCGCCACCATCAGCGTCTT	RT-qPCR
OsIAA26-qRT-R	CCCGTCCATGCTCACCTTCA	RT-qPCR
OsGS9-qRT-F	GACGCAAGCAGCGACCAGCA	RT-qPCR
OsGS9-qRT-R	GCCTGGCAGTTGGAGGATGAGC	RT-qPCR
OsGW7-qRT-F	TGGAGGAAGAACAAGAAC	RT-qPCR
OsGW7-qRT-R	ATATGACGTGTCGAGGAC	RT-qPCR
OsIAA19-GFP-F	ATTTGGAGAGGACAGGGTACCATGCCGCCGCCGCTCGAA	Subcellular localization
OsIAA19-GFP-R	CACCATGGTACTAGTGTCGACTTTGTTCTGACCTGTCTGTTCCAG	Subcellular localization
OsIAA19-F	GCACGCATGCTTACTTGGC	Sequencing
OsIAA19-R	CTCTCTCCTCCCCTTCTCCAA	Sequencing
